# 1749. Clinical Characteristics and Enteric Viral Detection Among Children with One Vomiting Episode in the Absence of Diarrhea

**DOI:** 10.1093/ofid/ofad500.1580

**Published:** 2023-11-27

**Authors:** Jacob D Johnson, Justin Z Amarin, Tess Stopczynski, Haya Hayek, Yasmeen Z Qwaider, Olla Hamdan, Laura S Stewart, Rendie McHenry, Aron J Hall, Daniel C Payne, Mary Wikswo, John R Dunn, Andrew J Spieker, James Chappell, Natasha B Halasa

**Affiliations:** Vanderbilt University Medical Center, Lexington, Kentucky; Vanderbilt University Medical Center, Lexington, Kentucky; Vanderbilt University Medical Center, Lexington, Kentucky; Vanderbilt University Medical Center, Lexington, Kentucky; Vanderbilt University Medical Center, Lexington, Kentucky; Vanderbilt University Medical Center, Lexington, Kentucky; Vanderbilt University Medical Center, Lexington, Kentucky; Vanderbilt University Medical Center; Division of Pediatric Infectious Diseases, Nashville, Tennessee; Division of Viral Diseases, National Center for Immunization and Respiratory Diseases, CDC, Atlanta, Georgia; US Centers for Disease Control and Prevention, Atlanta, GA; Centers for Disease Control and Prevention, Atlanta, Georgia; Tennessee Department of Health, Nashville, Tennessee; Vanderbilt University Medical Center, Lexington, Kentucky; Vanderbilt University Medical Center, Lexington, Kentucky; Vanderbilt University Medical Center, Lexington, Kentucky

## Abstract

**Background:**

Clinical presentations and etiologies of acute gastroenteritis (AGE) are variably captured by current definitions. Nonspecific presentations of AGE, such as a single vomiting episode within 24 hours without diarrhea, are common. We described clinical characteristics and viral detection among children presenting with one vomiting episode without diarrhea, comparing them to those presenting with multiple episodes or those who were asymptomatic.

**Methods:**

We conducted population-based surveillance of children presenting to Monroe Carell Jr. Children's Hospital at Vanderbilt as part of the New Vaccine Surveillance Network (12/01/2012–01/30/2016). Eligible children were aged >14 days and < 18 years, had ≥1 vomiting episode or ≥3 episodes of loose stool within 24 hours, and presented within 10 days of symptom onset. Additionally, we recruited asymptomatic children and collected stool samples within 5 days of enrollment. We tested stool samples by enzyme immunoassay for rotavirus and by reverse transcription polymerase chain reaction assays for rotavirus (confirmatory), norovirus, sapovirus, and astrovirus. We restricted our analysis to children without diarrhea. For comparisons, we used Pearson’s *χ*^2^ test and the two-sample *t*-test with unequal variances for categorical and continuous variables, respectively.

**Results:**

Our final study population included 180 children with one vomiting episode, 853 children with multiple episodes, and 1,255 asymptomatic children. Demographic and clinical characteristics of the children are compared in **Table 1**. At least one virus was detected in 32 children with one vomiting episode (17.8%; reference), 364 children with multiple episodes (42.7%; *p* < 0.001), and 212 asymptomatic children (16.9%; *p* = 0.77). Norovirus was the most common virus detected across all three groups, and children with one vomiting episode were more likely to present with fever than those with multiple episodes.

Table 1

**Figure:**
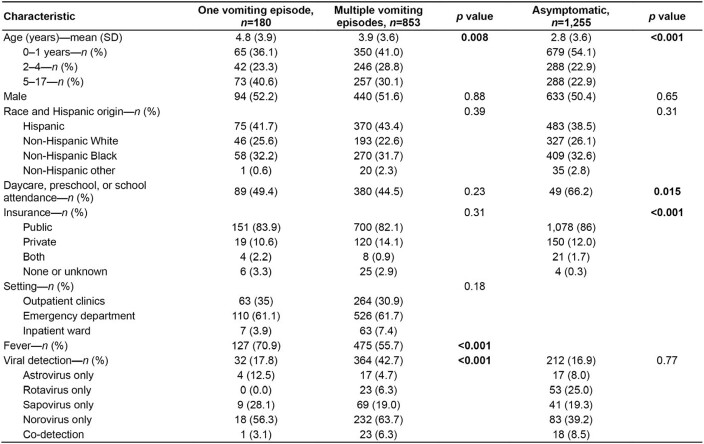

Clinical characteristics and viral detection among children presenting with one vomiting episode without diarrhea, those presenting with multiple episodes without diarrhea, and those who were asymptomatic.

**Conclusion:**

Viral detection in children presenting with one vomiting episode was comparable to that in asymptomatic children and considerably less frequent than in children with multiple vomiting episodes. Our findings suggest that one vomiting episode without diarrhea is not a comparatively strong indicator of viral acute gastroenteritis.

**Disclosures:**

**Natasha B. Halasa, MD, MPH**, Merck: Grant/Research Support|Quidell: Grant/Research Support|Quidell: donation of kits|Sanofi: Grant/Research Support|Sanofi: vaccine support

